# Oil Frying Processes and Alternative Flour Coatings: Physicochemical, Nutritional, and Sensory Parameters of Meat Products

**DOI:** 10.3390/foods13040512

**Published:** 2024-02-07

**Authors:** Luzia Ellen de Mendonça Lima, Bruna Leal Lima Maciel, Thaís Souza Passos

**Affiliations:** 1 Department of Nutrition, Health Sciences Center, Federal University of Rio Grande do Norte, Natal 59078-970, RN, Brazil; luziaellen29@gmail.com; 2 Nutrition Postgraduate Program, Health Sciences Center, Federal University of Rio Grande do Norte, Natal 59078-970, RN, Brazil; bruna.maciel@ufrn.br

**Keywords:** frying, nonconventional flours, moisture retention, oil absorption

## Abstract

The frying process changes can be desirable and undesirable, involving the physicochemical, nutritional, and sensory aspects, depending on the food and oil properties and the frying process. In this context, alternative flours emerge as a strategy for adding value to the food since they are rich in fiber, vitamins, and minerals, contributing to the variability of ingredients and the full use of food, including residues such as seeds and husks. This narrative review aims to gather current scientific data addressing the alternative flour coatings on breaded meat, mainly chicken, products to evaluate the effects on fried products’ nutritional value, physicochemical parameters, and sensory attributes. Scopus, Science Direct, Springer, and Web of Science search bases were used. This review showed that alternative flours (from cereals, legumes, fruits, and vegetables) used as coatings increase water retention and reduce oil absorption during frying, increase fibers and micronutrient content, which are not present in sufficient quantities in commonly used flours due to the refining process. These flours also reduce gluten consumption by sensitive individuals in addition to favoring the development of desirable sensory characteristics to attract consumers. Therefore, frying processes in oil promote a reduction in humidity, an increase in oil absorption and energy content, and a decrease in vitamin content. In this context, coatings based on alternative flours can reduce these adverse effects of the frying process.

## 1. Introduction

Frying is a culinary technique that consists of preparing food in oil at high temperatures, which acts as a means of mass and heat transfer, causing changes that characterize the final product. It is one of the oldest cooking methods, known and used worldwide for its practicality [1,2]. It can occur by partial (shallow frying), where the food is fried in an amount of oil that is not enough to cover the food, or total (deep frying) immersion of food in oil, where the amount of oil used must completely cover the food [3,4].

When the food absorbs oil, aspects related to the quality of the food itself change. In this sense, the reactions between the food and the oil during the frying process can cause changes in the final product [5,6]. Oil absorption directly impacts the increase in energy, one of the most significant transformations related to the frying process. Several factors related to the process and/or food, such as water content, crust microstructure, product geometry, frying time, temperature, and oil quality, can influence oil absorption [7,8,9]. The changes during frying affect the physicochemical, sensory, and nutritional parameters of food quality [10]. Therefore, understanding the mechanisms of oil absorption during frying is essential for developing alternatives that promote reducing fat content while maintaining the favorable characteristics of these foods. Thus, there is a growing interest in new strategies to reduce oil absorption in fried foods [11].

Coating of fried foods is an effective strategy for reducing oil absorption [12]. Wheat flour is the most used flour for this purpose, but there are many alternative flour sources that have a dense nutritional composition, a range of health-promoting bioactive compounds, and dietary fibers with diverse structures [13].

Dietary fibers are considered functional because they offer several health benefits in addition to the nutritional value inherent to their chemical composition, and they may play a beneficial role in reducing the risk of chronic degenerative diseases. Therefore, demand for these foods is growing [14,15]. Thus, alternative flours of cereals, legumes, fruits, and vegetables added in culinary preparations can be included as an ingredient in the diet, being a way to encourage healthy eating habits and a strategy for the development of new products with beneficial effects on health and increasing the possibilities of partial replacement of refined wheat flour with alternative flours [16].

Since oil absorption is a surface phenomenon [17], several studies have been conducted to reduce this problem using different coating materials. The breading process essentially refers to the coating applied to the outer surface to provide the food’s crispness, flavor, color, and appearance [18,19]. In addition, coatings can change the microstructure of the crust, forming a physical barrier against oil absorption [12]. Different coating formulations can be used in meat products to improve sensory attributes, provide visual and structural qualities, and modify the amount of fat absorbed during frying. Wheat flour is the main component of most coating batters, but alternative flours can also be used to provide different flavors or textures [20].

Considering the importance of discussing the use of other types of coatings based on alternative flours, a narrative review was carried out to gather scientific data available about the use of flour obtained from cereals, legumes, fruits, and vegetables as coatings for meat products to evaluate the effects promoted in the moisture and lipid content, oil absorption, and the nutritional and sensory quality of the final product. An electronic search was conducted from 2021 to 2023 in Scopus, Science Direct, Springer, and Web of Science databases. The selection criteria were as follows: the manuscripts should be in English, available in full in the cited databases, and published in the last two decades. Descriptors and related terms were inserted in databases, and the papers involving vegetable flour coatings, fried meat products, and the aspects involving physicochemical, nutritional, and sensory parameters were selected.

## 2. Oil Frying Process

Frying is a typical process used for a long time in food manufacturing. Different peoples over the centuries have consumed various fried products. Knowledge about the frying process was common in the 14th century [10]; it is believed to have been first performed by the ancient Chinese. Still, some authors suggest that the current frying process arose and developed near the Mediterranean area under the influence of olive oil [20,21].

The technique of frying is simple, quick, and low-cost. Nowadays, it is employed in restaurants, the food industry, and households due to the characteristics acquired by fried foods [22,23]. In the frying process, food is cooked in oil at temperatures above boiling. Specific conditions such as temperature, pressure, utensils, food, and frying oil are identified as components of this process [20].

Several phenomena, such as heat, moisture, and oil transfer, coincide during the entire process. Interactions between oil and food promote chemical reactions capable of modifying the sensory and nutritional characteristics of the food during the frying process [10]. In addition, the oil used also undergoes constant changes. Therefore, to avoid the degradation of the oil, the frying temperature should not exceed the temperature of 180 °C [24,25].

At the end of the frying process, fried preparations have a high oil content and an increased lipid and energy content. However, these products have high palatability, which makes them very popular. Thus, a wide variety of fried products has been developed [22,26].

### 2.1. Types of Oil Frying

Two types of frying are commonly used in food preparation, superficial, or shallow frying, and deep fat frying. The techniques differ in the amount of oil used and the heat transfer method [3,4,24].

Shallow frying is indicated as most suitable for foods with a large contact surface [3]. In this case, the food is placed in a frying pan containing a small amount of oil, which can vary according to the irregularities of the food’s surface. This layer of oil is responsible for transferring heat to the surface of the food, which, in this case, happens by conduction [10,24]. On the other hand, in this type of frying, this transfer is not stable in all parts of the food, requiring it to be turned over for even cooking [24].

The high temperature in the surface layer is responsible for the food’s water loss, causing temperature variations and the formation of the distinctive color characteristic of these products, resulting from the Maillard reaction [3,24]. Time and temperature must be carefully monitored to prevent food burning. Shallow frying is commonly used to prepare fish, meat, and various vegetables [20].

Deep frying, widely used domestically, commercially, and industrially, plays an important role in the market [27]. It is considered a rapid dehydration process in which water is removed from food by fast heating in oil. The process is characterized by total immersion in oil, depending on the product and the desired result. The food is subjected to a temperature varying from 150 to 190 °C [10,24]. In this case, heat and mass transfer occur continuously and simultaneously.

Heat transfer can occur by convection between the oil and the food surface and conduction within the food structure [10]. Deep frying aims to produce food for immediate consumption or further processing [21]. This process allows foods to be fried until desired properties, such as colors and flavors, are achieved [20].

It is worth highlighting that this narrative review focused on conventional frying methods, shallow frying, and deep frying, but it is known that there are other methods, such as air-fryer cooking. However, as it is a new method, there are few published studies related to meat products and comparing physicochemical, nutritional, and sensory aspects with conventional methods. According to the literature, air fryers appear similar to pan frying without oil in nutritional and physical characteristics due to a longer cooking time and because they appear healthier than pan frying with oil [28].

### 2.2. Mechanisms Related to Oil Absorption

**Figure 1:**
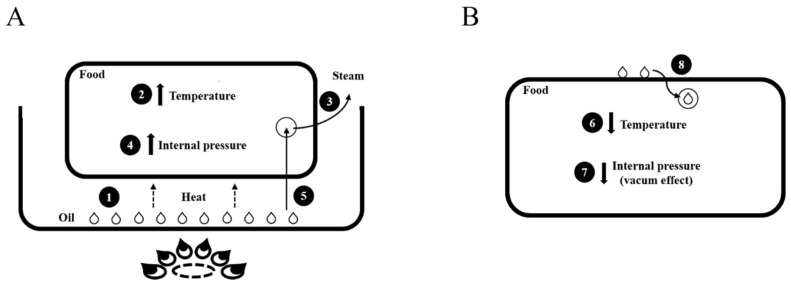
Oil absorption mechanisms. (**A**) During frying: (1) heat transfer from oil to food; (2) temperature rise; (3) loss of water in the form of steam; (4) increased internal food pressure; (5) oil inlet, replacing water. (**B**) After frying (cooling): (6) temperature reduction; (7) reduction of the internal pressure of the food and formation of the vacuum effect; (8) absorption of the oil present on the surface of the food. Based on Brannan et al. [17].

One of the most significant changes resulting from the frying process is the amount of oil absorbed by the food. Over the years, several studies have analyzed the mechanisms of oil absorption in different foods [6,29]. Some theories that can describe these mechanisms start from the idea that the oil migration into the food occurs through empty pores or capillaries in the food’s substrate and crust (Figure 1) [8,17,22].

Brannan et al. [17] described three mechanisms considered to be the main ones in oil absorption: (1) water replacement, (2) cooling phase effect, and (3) surfactant theory.

Water replacement (Figure 1A) also occurs during the frying process when the high temperature promotes the evaporation of the water contained in the food, generating a positive pressure gradient that replaces evaporated water with oil [30]. First, the water from inside the food migrates to the surface and then evaporates, allowing the crust to remain permeable. This process favors oil entry into the food to replace the water. However, the oil cannot penetrate the food easily during frying due to the constant vaporization and pressure buildup within the food [10].

The cooling phase effect (Figure 1B) occurs after the frying process is completed when the food is removed from the frying medium and loses heat. At this point, the steam reduction, with a consequent reduction in the internal pressure, results in a “vacuum effect”, causing the oil trapped on the surface to penetrate through the pores while the food is cooling and vaporizing is decreasing [17]. In addition, when food is removed from the hot oil, the temperature difference causes an increase in capillarity, causing the oil to advance into the open pore spaces [8]. This phenomenon, known as the surface phenomenon, happens during the food cooling process. It is also believed to be responsible for the balance between adhesion, which is the oil’s ability to cling to the surface of the product, and oil draining when the food is removed from the frying medium [8,31].

Also known as the solidification mechanism, the oil’s ability to adhere to the product happens during cooling. As the temperature decreases, the oil viscosity increases, which leads to greater adhesion to the surface of the food. This solidification starts immediately after the product is removed from the hot oil [29].

The surfactant theory is based on hydrolytic reactions caused by high temperature and water loss during the frying process. These reactions are responsible for forming surfactants and polar compounds that accelerate oil degradation and reduce the interfacial tension between oil and food. Thus, the contact between the two increases, causing excessive oil absorption [17,30].

Although the latter theory was discarded by Dana and Saguy [30], it is believed to be explained by evidence that oil absorption during frying largely depends on the contact angle and interfacial tension of oil and water. Surfactants are also related to differences in the surface and interior of the food caused by aged oil. As the contact time between the two increases, more heat is passed to the food, resulting in greater surface dehydration and water migration from the interior to the outside of the food. Thus, high concentrations of surfactants result in oil-soaked products with an over-fried exterior and an undercooked interior [32].

Despite many theories, several factors are responsible for the transformations during the frying process. Changes in the product and the oil quality further complicate the phenomenon of oil absorption, which remains a complex mechanism not yet fully understood [33].

### 2.3. Factors That Influence Oil Absorption

#### 2.3.1. Food Properties

Size, shape, contact surface, and composition of the food are conditions that can determine the amount of oil absorbed. In addition, the mass transfer phenomenon is responsible for replacing water with oil in food. Therefore, the amount of water strongly correlates with oil absorption [11,33]. Water evaporation occurs on the surface resulting in structural changes, such as dehydration of the crust and formation of pores, leading to the increased entry of oil into the food [29]. On the other hand, the contact surface and the product roughness are associated with surface permeability since these characteristics allow greater adherence of oil to the crust [11].

It is believed that the initial amount of water and solid content in the food are factors that can influence the absorption of oil [33]. However, oil absorption appears to be more closely related to moisture loss than to initial moisture, with the amount of oil absorbed being directly proportional to the moisture lost [7]. Furthermore, it has been suggested that oil absorption is greater when the contact surface increases and the product thickness decreases. This may be related to the greater amount of water in the food and, consequently, more significant oil transfer, given the need for a longer frying time [33].

#### 2.3.2. Oil Properties

During the frying process, the oil can change due to high temperature and the presence of oxygen, which can directly affect the physical properties of the oil, such as viscosity, interfacial tension, color, and density [21,22]. In addition, thermal and oxidative degradation can occur, promoting the formation of volatile and nonvolatile products, which will compromise food quality due to contamination by toxic substances, sensory changes, and increased oil absorption [34].

The type of oil used also influences oil uptake by fried products; however, it may be more related to the quality of the oil [7,35]. Studies claim that the amount of unsaturated fatty acids in the oil can influence its absorption by food and that the fatty acid composition of oils is highly related to their viscosity and surface tension, affecting the wettability of oil and food during the frying process, heat transfer and mass transfer rate, drainage of oil during the post-frying and cooling phases, and, therefore, the oil content absorbed by the final products [36].

Higher viscosity makes it harder for oil to drain from the product’s surface. Viscosity increases with the formation of dimers and polymers in aged oils and a decrease in the contact angle due to the formation of polar compounds [7]. The increase in viscosity can contribute to the increasing amount of oil on the surface of the food. In contrast, the decrease in the contact angle could increase the oil’s wetting properties, resulting in a higher content of absorbed oil [21,37].

Oil reuse is one of the factors that increase oil viscosity during frying. In addition to viscosity, other physical changes caused by repeated heating of the oil are the darkening of color, which occurs due to the development of pigments during oxidation, and the thermal decomposition of fatty acids that diffuse into the oil during frying and foaming. Moreover, other chemical changes, such as an increase in free fatty acids, are due to the cleavage and oxidation of double bonds to form carbonyl compounds and low molecular weight fatty acids [38]. Studies have shown that the oil and the food can deteriorate throughout the frying oil reuse cycles, reducing healthy components such as omega 3, omega 6, cis, and vitamin A [39].

#### 2.3.3. Frying and Pos-Frying Properties

The frying temperature is one of the most critical parameters. Ghaderi, Dehghannya, and Ghanbarzadeh [40] concluded that a higher temperature used in the frying process (up to 190 °C) reduces oil absorption. This increase in temperature also promotes physicochemical transformations that accelerate the degradation of the oil. Despite this, oil degradation increases viscosity, resulting in greater adhesion to the surface, consequently hindering the drainage process during the cooling time and preventing oil entry into the food [21].

Other aspects are related to post-frying conditions, precisely, the cooling process. For example, oil absorption might decrease if the food is taken out of the oil while the temperature is still rising. Likewise, if the food is shaken vigorously immediately after the oil is removed, absorption can be limited due to the draining of the liquid oil on the product’s surface [41].

### 2.4. Alteration of the Physicochemical, Sensory, and Nutritional Properties of Foods Related to the Frying Process

Many physicochemical transformations occur with foods during the frying process, promoting structural changes at macro and micro levels and contributing to fried products’ unique flavor and texture [10]. The chemical composition determines aspects of the product, including sensory and nutritional characteristics [20]. The effect of frying on food involves the oil, which influences the quality of the food, and the direct impact of temperature on the fried product [3]. The heat transfer during frying is responsible for foods’ protein denaturation, gelatinization, and water vaporization. The mass transfer is characterized by the loss of starch, soluble matter, and water, with consequent diffusion of oil into the food [8].

The nature and rate of product decomposition depend, among other things, on the composition and time of oil use, type of oil, temperature, and duration of the frying process, and the characteristics of the food to be fried [3]. The hydrolysis, oxidation, and polymerization processes that occur with the oil produce volatile and nonvolatile compounds that give the typical flavor of fried products, making them more attractive [7]. The fat used in frying interacts with food ingredients to develop the characteristic texture of fried foods, as well as help to transport, intensify, and release flavors [42]. As a naturally palatable agent, fat replaces water in fried foods, promoting a softening and wetting effect, enhancing the flavor, and contributing to the crispness of the product, making it more pleasant.

Browning, the crust formation, which results from dehydration of the food surface, and the formation of components that characterize the flavor of fried foods occur during the frying process and develop from combinations between the Maillard reaction and the absorption of oil components [3]. The characteristic flavor results from the lipid degradation products originating from the frying oil. The attractive golden-brown color relates to products undergoing frying at the ideal temperature and time conditions. At the same time, those with a light golden color may have been fried at a lower temperature in a shorter time than ideal and may be undercooked inside [42]. In addition to color, the Maillard reaction in these foods is also responsible for aroma, flavor, and texture [43].

The frying process can also change the nutritional composition of foods, leading to increased energy and loss of essential micronutrients. The protein content of foods is usually increased during frying due to dehydration [5]. The increase in food energy is one of the main reasons for concern about fried food, and this happens due to the absorption of oil from frying when oil replaces the spaces left by the loss of moisture [44]. In addition, the change in lipid composition may be a much more critical factor. The formation of trans-fatty acids is very common in producing these foods, and this lipid fraction is related to an increased risk of cardiovascular diseases. Likewise, potentially carcinogenic compounds have been identified [5].

Aladedunye and Przybylski [45] evaluated the effect of frying temperature on canola oil degradation. They observed a significant increase (*p* < 0.05) in the content of total polar compounds and trans-fatty acids as a function of temperature and frying time. The polyunsaturated fatty acids in the oil were reduced in direct proportion to temperature and frying time. The thermal and oxidative degradation rates were dramatically higher at the highest temperature tested (215 °C), forming greater amounts of potentially harmful components. The authors concluded that raising the frying temperature above 195 °C may cause the isomerization of polyunsaturated fatty acids. The number of trans isomers may increase in such a way as to nullify the claim that fried products are free of trans fats.

On the other hand, when heated to high temperatures, oils can undergo reactions such as hydrolysis, oxidation, thermal-oxidative changes, and polymerization, which affect the quality of the oil and, consequently, the quality of fried foods [38,46]. Qualitative changes in the frying oil, which may indicate these reactions, can be visually perceptible through the intensification of color, formation of smoke, and consequent formation of compounds harmful to health, such as acrolein, and an increase in viscosity [47].

About the micronutrients, the amount of minerals appears to be preserved and, in many cases, may increase due to the concentration of nutrients. On the other hand, vitamins are thermosensitive, and loss can occur through oxidation, with some vitamins and antioxidant compounds being eliminated in the process. Oil absorption can also affect the composition, texture, size, and shape of the food, resulting in a loss of nutrients, especially vitamins [11]. Many vitamins are sensitive to oxidation and high temperatures. Still, this loss may depend more on the type of food and the internal temperature as higher temperatures reach the surface of the food. During frying, the oxidation of unsaturated fatty acids leads to the loss of vitamin E. Vitamin A can be lost to the oil during the process, which can vary with the frying time [48].

Piñeiro et al. [49] evaluated the effects of salting and frying on the content of water-soluble (B2, B3, B9, and B12) and fat-soluble (A, D, and E) vitamins in swordfish (*Xhiphias gladius*). The samples were fried in olive oil at 120 °C for 7 min. The authors observed that, due to the loss of water from the samples during salting, there was a significant decrease in the values of vitamin B2, while the content of the other vitamins remained constant. However, water-soluble and fat-soluble vitamins showed great thermolability in the frying process, with high retention percentages ranging from 50 to 100%. Vitamins B2 and B3 showed stability during frying, while vitamin B9 showed a loss of about 80%. Vitamin B12, on the other hand, showed a loss after salting and an increase after the frying process. Among the fat-soluble vitamins, vitamin A was significantly reduced after frying, while vitamins D and E remained stable. The stability of the vitamin E content after frying can be explained by olive oil, which is rich in this vitamin.

## 3. Coatings Based on Flours from Plant Sources

The coating or breading of foods has been studied as an alternative for reducing oil absorption in fried foods. Some researchers have already observed that using these coatings can positively reduce the oil absorbed in the frying process. These products give a crispy outer surface with an attractive color and act as a protective layer, preserving the food’s moisture and flavor [21].

The food coating system can be divided into batter and breading [43]. The batter is a liquid mixture composed of water, flour, starch, and seasonings, in which the food is immersed before the frying process to form a crispy crust, leaving the fried food with a unique flavor [50]. It can be used as a single coating or combined with breading, acting as an adhesion coating between the food and the coating flour [43]. Based on the literature, the thickness of the coating layer is crucial as it is directly related to sensory acceptance by consumers. More dense dough generally contains a higher percentage of flour and, thus, a greater coating pickup. Furthermore, batter consistency is an essential quality attribute of coatings, influencing their performance during frying [43,51].

Breading can be defined as the coating applied to a previously moistened or batter-coated food. It is a type of flour, usually derived from cereals, which may or may not be seasoned [50]. Traditionally, this type of coating is mainly composed of wheat flour or a product derived from cereal flour, such as breadcrumbs [43]. These flours, however, are rich in gluten, which can retain gases and expand during frying, producing a cellular structure and providing a spongy, porous, and desirable coating, essential for a crispy texture, but which also facilitates moisture loss and absorption of oil [51]. However, other types of flour derived from grains, such as rice and corn, can also be used as breading. Flours based on other plant sources, such as soybeans, potatoes, chestnuts, cassava, and almonds, are used to add value or have a lower carbohydrate content and a unique texture. Because they do not contain gluten in their composition, they are alternatives for individuals with celiac disease or non-celiac gluten sensitivity [43].

In addition to people who have food intolerance or food allergies, there has been an increase in consumer awareness about the relationship between nutrition and health and, consequently, an increase in demand for health-promoting foods in recent years [52]. Alternative flours are ingredients rich in functional compounds that are intended to produce a positive effect on consumer health [53]. In this context, fruit and vegetable by-products, such as pomace, peel, pulp, and seeds, are good sources of phytochemicals, presenting antioxidant, anticancer, hypoglycemic, antimicrobial, cardioprotective, neuroprotective, anti-inflammatory, and immunomodulatory properties [52]. In meat products, the addition of vegetable products can improve the functional properties and nutritional and sensory qualities of the final products [54,55].

### 3.1. Cereals

Most breaded foods are made from processed cereals, and many studies have evaluated the effects of using flours produced from grains (Table 1). Gamonpilas et al. [56] investigated the impact of cross-linked tapioca starches on coating dough viscosity and oil absorption in breaded chicken strips. The chicken strips were breaded and fried in palm oil at a temperature of 180 °C for five minutes. The use of cross-linked tapioca starches in the dough significantly reduced the oil content on the surface of the samples. This reduction was attributed to the extent of cross-linking of the starch replaced by wheat flour. Cross-linking within the starch granules can make them more resistant to deformation during heating, promoting a barrier against oil. Thus, the usefulness and feasibility of using cross-linked tapioca starches in reducing oil absorption in fried products are highlighted.

Park et al. [57] analyzed the influence of the addition of wheat fiber on the physicochemical properties and the sensory characteristics of pork loin chops. The formulations were produced with different concentrations of wheat fiber (0, 1, 2, 3, and 4%) and the breaded samples were fried in soybean oil at 170 °C. The fat content and, therefore, energy decreased significantly with increasing levels of wheat fiber. It is believed that the hydrating properties of dietary fiber promoted a more substantial binding power with moisture than with fat so that the moisture content was preserved and the absorbed fat content decreased. Trained panelists carried out the sensory evaluation for attributes such as color, flavor, softness, juiciness, and overall acceptance. Samples with added wheat fiber received higher evaluations for color, softness, juiciness, and general acceptance than the control group. In terms of color, samples with 1% and 2% dietary fiber received better evaluations, while tenderness, juiciness, and overall acceptability received excellent ratings as the amount of wheat dietary fiber added increased. The group treated with 3% wheat fiber received the highest score for overall preference.

Dogan, Sahin, and Sumnu [58] compared the effects of adding different flours, including rice flour, on the quality of deep-fried chicken nuggets, using sunflower oil at 180 °C, for 3, 6, 9, and 12 min. Although it did not significantly affect moisture retention, the addition of rice flour in the formulation significantly reduced oil absorption in the samples when compared with the control (wheat and corn flour).

Nasiri et al. [59] evaluated the effects of adding different flours to the coating batter, using soy and corn flour added to wheat flour, in proportions of 5 and 10%, to coat samples of shrimp nuggets (*Penaeus* spp.), which were pre-fried at 150 °C in sunflower oil for 30 s and frozen for a week. The samples were thawed at 4 °C for 24 h and then subjected to the frying process for 0, 60, 120, 180, 240, and 300 s at 150, 170, and 190 °C. Formulations containing corn flour showed higher oil absorption than those containing soy flour and control (wheat flour). In addition, among all the formulations tested, the formulation containing 5% of corn flour had the lowest moisture content. These results may be related to the viscosity of the dough containing corn flour, which did not differ from the control when 10% was added and decreased when 5% of corn flour was added. This way, the lower capacity of the dough to bind to water results in greater availability of water to be evaporated during frying and, consequently, greater oil absorption.

Ketjarut and Pongsawatmanit [60] investigated the effects of the partial replacement of wheat flour (WF) by tapioca starch (TS) on the quality of breaded and fried chicken wings. Tapioca starch was added to the batter in 25 and 50% proportions. The samples were breaded and then pre-fried in refined palm oil for 4 min at an average temperature of 167 °C using a deep fryer. Part of the samples was taken to the oven at 195 °C for 8 min, and after cooling, they were packaged and frozen (−18 °C) for two weeks. The final frying was performed at an average temperature of 195 °C for 3 min. A decrease in oil content in the crusts (49.7–41.3%) was observed as the addition of TS increased. The crust of the dough containing only WF showed larger pores in the pre-fried product due to the thermosetting gluten network, facilitating oil entry into the crust layer. For sensory evaluation, the samples were randomly presented to fifty untrained panelists, one by one, immediately after frying. Appearance, color, crust thickness, adhesion between the crust layer and the chicken, crispness, and overall acceptance were evaluated using a nine-point hedonic scale. It was observed that the notes increased with increasing TS substitution in the WF/TS flour blends, and the sample containing 50% TS was the one that presented better results for all the attributes evaluated.

Kilinççeker [61] evaluated the use of oat flour as a coating material in fried chicken meatballs, replacing wheat and corn flour with oat flour in proportions of 3:1, 1:1, and 1:3 (*w*/*w*) wheat: oats in batter and 3:1, 1:1 and 1:3 (*w*/*w*) corn: oats in breading. Formulations without adding oats were used as a control, and all samples were fried for 5 min at 180 °C. The moisture content in the fried samples decreased with increasing oat flour in the dough, while the lipid content increased. The amount of gluten was reduced as the oat flour content in the dough increased. Thus, water loss and increased oil absorption may be related to gluten reduction since the structure formed by gluten offered more resistance to mass transfer in the samples. Despite this, the use of oat flour positively affected sensory properties such as appearance, color, and flavor. Ten trained judges and a nine-point hedonic scale were used for sensory evaluations of appearance, color, odor, taste, and texture. Results showed that all the breading mixes with oat flour had a higher appearance, color, taste, and overall acceptability scores than the control. The results showed that oat flour has functional and nutritional properties and can be used in coating formulations.

### 3.2. Legumes

Legumes are plants of the Fabaceae (or Leguminosae) family or the fruit of these specific plants. They are characterized by seeds carried in pods and are often edible, which form part of the diet. Nutrient content includes complex carbohydrates, low fat, proteins with a good amino acid profile, vitamins such as B complex, folate, ascorbic acid, and vitamin E, and minerals, including calcium, copper, iron, magnesium, phosphorus, potassium, and zinc, as well as antioxidants, polyphenols and several other phytochemicals with biological activities important to human health [68]. As a result, legume flours are an interesting strategy for breading meat products and have been evaluated through some studies (Table 1).

In a study that evaluated the use of chickpea flour in the coating of chicken nuggets, previously covered with batter, fried in hydrogenated palmolein margarine at 180 °C, Kilinççeker and Kurt [63] found that in addition to improving sensory properties, coated samples that contained a greater amount of chickpea flour in the batter showed the highest moisture preservation and the lowest fat content. The authors concluded that this result could be attributed to the higher adhesion effects of chickpea flour on the batter since its moisture content is lower and its protein content is higher, which strengthened the batter’s structure coating and prevented moisture migration from the nuggets. As for the sensory analysis, ten trained judges assessed the sensory properties using an eleven-point hedonic scale for appearance, color, odor, taste-flavor, and texture. The increasing chickpea flour in the mixtures increased the scores of sensory parameters. All sensory properties in the coated nuggets were at high acceptable levels. The increasing sensorial notes were related to the golden color, pleasant fried and soft chickpea odor, taste, and texture.

Kilinççeker, Hepsağ, and Kurt [64] evaluated the potential use of lentil (L) and chickpea (C) flours as coating materials in fresh and frozen chicken meatballs fried in sunflower oil. The flours were mixed in different proportions (2:1, 1:1, 1:2 L:C *w*/*w*), and corn flour was used for the control samples. The authors observed that after the frying process, flour mixtures in the proportions of 2:1 and 1:1 L:C (*w*/*w*), despite not showing a significant difference, promoted greater retention of humidity than the control samples, thus being good options for the coating material. The sensory analysis also did not show a statistically significant difference (*p* > 0.05). Samples were served randomly to ten trained panelists, and the sensory properties were assessed using a nine-point hedonic scale for appearance, color, odor, flavor, and texture. Sensory scores of deep-fat-fried meatballs coated with lentil and chickpea flour were acceptable.

Nasiri et al. [59] also evaluated the effects of using soy flour to coat shrimp nuggets (*Penaeus* spp.). The coating promoted greater moisture retention and less oil absorption during frying. Water retention was attributed to the high water-binding capacity presented by soybean flour. In contrast, the lower oil absorption was attributed to the higher protein content, higher water-binding capacity, and higher viscosity of the flour, which favored the control of the loss of moisture and, consequently, the absorption of oil during frying.

Dogan, Sahin, and Sumnu [58] evaluated the effects of adding soy flour on the quality of deep-fried chicken nuggets, using sunflower oil at 180 °C for 3, 6, 9, and 12 min. After frying, soybean flour promoted higher moisture content due to a more resistant crust that served as a barrier to water loss. The texture of fried products was also significantly better when using soy flour (*p* < 0.05). The authors concluded that due to its high water-binding capacity, the batter with the addition of soy flour could control the loss of moisture and, therefore, the absorption of oil during frying. In addition, the higher viscosity of the batter with soy flour increased adhesion in the samples, which was also effective in controlling oil absorption.

Para et al. [65] investigated the influence of black bean flour as a coating material on the physicochemical properties of chicken nuggets, using batter formulations prepared with the flour at concentrations of 25 and 35%. The samples were covered with the batter and fried in a fryer with refined cottonseed oil for 3–5 min at 175 °C. Characteristics such as coating thickness, cooking yield, crude protein, ethereal extract, ash, and crude fiber content of the nuggets increased significantly (*p* < 0.05) with the increase in flour. The pH and the moisture/protein ratio decreased significantly (*p* < 0.05). The ether extract of the formulation containing 35% (17.43%) of the flour was higher than the formulation containing 25% (16.49%), which may be due to the greater absorption of oil by the sample since the flour content is higher. Black bean flour provided higher scores for the sensory attributes of color and appearance, flavor, texture, and general acceptability, showing a significant difference (*p* < 0.05) for all but juiciness. Color and appearance scores, flavor, texture, and overall acceptability decreased significantly as the level of black bean flour increased in the batter mix. This may be due to the intensity of the dark pigmentation of black bean flour and the greater proportion and a thick coating of flour, masking the product’s flavor. These results were better when the flour was used at a concentration of 25%. This proportion is considered optimal in the preparation of chicken nuggets.

The use of sorghum (*Sorghum bicolor*), millet (*Pennisetum glaucum*), and soybean (*Glycine max*) flours in deep-fried chicken breast fillet coating were evaluated by Kwaw et al. [62]. The fillets were breaded with the flours, used individually, and fried in sunflower oil at 175 °C. After frying, when comparing the samples coated with flour individually and the control sample breaded with wheat flour, the samples coated with legume flour had a lower fat content. This result may be due to an increased porosity of the crust of the samples coated only with cereal flours, which promoted increased oil absorption. The percentage of losses caused by frying was related to moisture loss and oil absorption, and samples breaded with soy flour (16.73%) had the lowest percentages after the analyses, which may be related to protein denaturation. The results indicated that legume-based flours developed more substantial barrier properties against moisture losses and, thus, low percentages of losses by frying. A sensory test was also conducted with 45 untrained panelists using a nine-point hedonic scale to evaluate color, flavor, moisture, texture, aroma, and overall acceptability. The sample breaded with soy obtained better ratings for moisture, compared with cereal flours, and for texture, compared with the uncoated sample. Crust color evaluation revealed that the sample breaded with soy was the most preferred among samples coated with flour individually. The assessment of flavor, aroma, and overall acceptability showed a similar trend with soy, with higher scores than samples breaded with cereals.

Considering the mixture of different sources of flour, there are few studies. Kwaw et al. [62] investigated flour mixtures prepared in proportions of 1:1 sorghum:soybean and millet:soybean (*w*/*w*), and 1:1:1 sorghum:millet:soybean (*w*/*w*/*w*), and samples were fried in sunflower oil at 175 °C. The percentage of losses caused by frying in samples breaded with a mixture of sorghum and soy flours showed the lowest percentages (20.88%) after analysis, which, as discussed previously, may be related to protein denaturation. The authors reported that samples coated with mixtures containing soy flour showed a slight decrease in fat content, which is associated with the properties of soy in the formulations. As for the sensory test, the samples coated with the composite flours received better moisture ratings than the individual flours. Sorghum–soy was the preferred breading mixture, and evaluation of flavor, aroma, and overall acceptability showed a similar trend with soy composite flours (except millet). Chicken breast coated with an equal proportion of soy and sorghum flour was the most preferred, with an overall acceptability of 84.44%, showing that this formulation could improve the quality and acceptability of chicken breast compared with conventional wheat flour. The results showed that combining soy and sorghum produced a composite flour positively impacting the breaded chicken’s characteristics.

### 3.3. Fruits and Vegetables

Fruits and vegetables are high in vitamins, minerals, and fiber. The total use of these foods can be an alternative, offering products of high nutritional value, developed from parts usually discarded, and contributing to reducing the negative impact on the environment [69]. Based on this, one of the ways to use it would be as flour obtained from these vegetables, with the potential for use in the coating of meat products (Table 1).

In a study that characterized the chemical composition of chicken nuggets breaded with pequi pulp flour in the breading layer, Braga-Souto et al. [66] used a control formulation composed of breadcrumbs and three formulations containing pequi pulp flour in proportions of 25, 50, and 100%. The samples were immersed in batter, pre-floured with the formulations containing pequi flour, immersed in batter again, breaded with cornmeal, and subjected to frying in an electric fryer with soybean oil at a temperature of 170 to 180 °C for 4 min. The pequi pulp flour added nutritional value to the breaded chicken, contributing to the lipid and protein content. Still, no significant differences (*p* > 0.05) were found between the moisture content and oil absorption of the formulations. Sensory analysis was conducted with sixty panelists who evaluated the samples based on appearance, texture, flavor, and overall acceptance characteristics using a nine-point hedonic scale. The results showed no statistically significant difference between treatments (*p* > 0.05), and the products were accepted. Pequi flour has the same potential for consumption and acceptance by the consumer as common breading, adding greater nutritional value.

Freitas et al. [67] analyzed the use of potato flour (*Solanum tuberosum* L.) cv. Monalisa in a mix of flour (corn, breadcrumbs, and potatoes) to cover the breaded chicken and compared it with commercial formulations. The chicken breast fillets were breaded with formulations containing the addition of 40, 60, and 80% of potato flour. Then, they were pre-fried and fried at 180 °C for 30 s and 3 min, respectively. The lipid content after the frying process was lower in the formulations with potato flour, decreasing as the amount of potato flour in the formulations increased. This result can be attributed to the smaller granulometry presented by the potato flour since the samples coated with flours with larger granulometry absorbed more oil. The sensory analysis was carried out to determine the proposed formulations’ overall acceptance. Fifty untrained panelists and a structured hedonic scale of nine points were used. The results showed that adding different levels of potato flour remained the same overall acceptance of the products.

**Figure 2:**
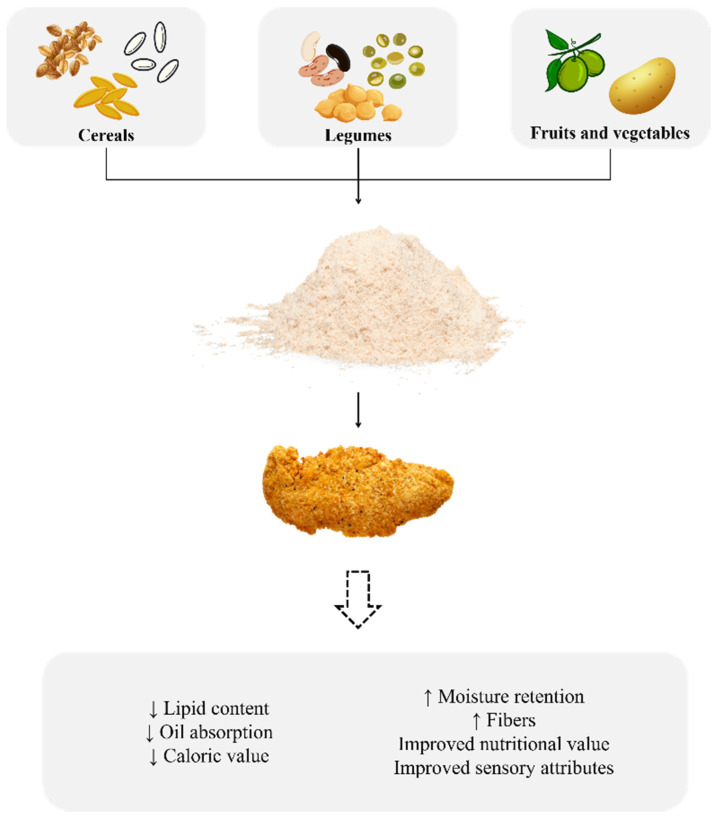
Benefits of using alternative flours. ↑: Increased; ↓: Decreased.

Thus, using vegetable flours, in addition to guaranteeing one of the main objectives of food coating, which is the improvement of sensory attributes, can improve the nutritional value and reduce oil absorption, which are directly related to the health of consumers and are reasons for concern regarding fried foods (Figure 2).

## 4. Conclusions

Coating foods with vegetable flours other than breadcrumbs and wheat can contribute to the reduction of oil absorption by meat foods during frying. The use of flours from different vegetable sources is a good alternative because they can add nutritional value, enhance fiber content, and promote the modification of the surface of meat foods, favoring the development of desirable sensory characteristics. The variety of sources of the flours presented in this study also effectively reduced the undesirable changes resulting from the frying process. Furthermore, they contributed to moisture preservation in these foods, reducing the caloric value, compared with breadcrumbs and wheat, and ensuring positive effects involving physicochemical, nutritional, and sensory properties.

## Figures and Tables

**Table 1:** Application of coatings based on flours from vegetable sources in deep-fried meat products.

Meat Product	Flour Source/ Proportion Used	Type of Oil	Frying Process Conditions	Results Obtained	References
Chicken strips	Cross-linked tapioca starches/20%	Palm oil	180 °C/5 min	↓ Oil absorption	[56]
Pork loin chops	Wheat fiber/1, 2, 3, and 4%	Soybean oil	170 °C	↓ Fat and calorie content; ↑ Fiber content	[57]
Chicken nuggets	Rice and soy flour/5%	Sunflower oil	180 °C/3, 6, 9, and 12 min	↓ Oil absorption	[58]
Shrimp nuggets	Soy and corn flour/5 and 10%	Sunflower oil	0, 170 e 190 °C/0, 60, 120, 180, 240, and 300 s	↑ Moisture retention; ↓ Oil absorption	[59]
Chicken wings	Tapioca starch/25 and 50%	Palm oil	Initial frying: 167 °C/4 min Final frying: 195 °C/3 min	↓ Oil absorption; improved sensory attributes	[60]
Chicken meatballs	Oat, wheat, and corn flour W:O/C:O—3:1, 1:1 e 1:3 *w*/*w*	Corn oil	180 °C/5 min	Improved sensory attributes	[61]
Chicken breast	Sorghum (*Sorghum bicolor*), millet (*Pennisetum glaucum*), and soy (*Glycine max*)/1:1 So: Mi, Mi:S, So:S (*w*/*w*) e 1:1:1 So:Mi:S (*w*/*w*/*w*)	Sunflower oil	175 °C	↓ Percentage of moisture loss	[62]
Chicken nuggets	Chickpea flour/1:3, 1:1, 3:1 *w*/*w* chickpea flour: corn, and 100% chickpea flour	Hydrogenated palmolein margarine	180 °C	↑ Moisture retention; ↓ Fat content	[63]
Chicken meatballs	Yellow lentil flour and chickpea flour/2:1 YL:CF, 1:1 YL:CF, 1:2 YL:CF	Sunflower oil	185 °C/6 min	↑ Moisture retention	[64]
Chicken nuggets	Black bean flour/25 and 35%	Cottonseed oil	175 °C/3–5 min	Improved sensory attributes	[65]
Chicken nuggets	Pequi pulp flour/25, 50 and 100%	Soybean oil	170 a 180 °C/4 min	Improved nutritional value	[66]
Chicken breast	Potato flour (*Solanum tuberosum* L.) cv. *Monalisa*/40, 60 and 80%	-	Pre-fry at 180 °C/30 s and fry at 180 °C/ 3 min.	↓ Lipid content	[67]

W: wheat; O: oat; C: corn; So: sorghum; Mi: millet; S: soy; YL: yellow lentil; CF: chickpeas. ↑: Increased; ↓: Decreased.

## Data Availability

The data are contained within the article. The data used to support the findings of this study can be made available by the corresponding author upon request.
